# Supplementary figures and images for: VGLL2 and TEAD1 fusion proteins identified in human sarcoma drive YAP/TAZ-independent tumorigenesis by engaging EP300
Source: eLife. 2025 May 8;13:RP98386. doi: 10.7554/eLife.98386 (PMC12061476; doi:10.7554/eLife.98386)

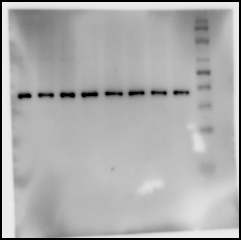

Supplement: Figure 1—source data 2. [file elife-98386-fig1-data2.zip › Figure 1, Source Data 2/GAPDH.png]

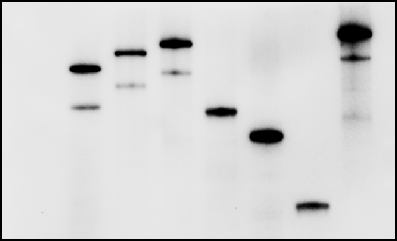

Supplement: Figure 1—source data 2. [file elife-98386-fig1-data2.zip › Figure 1, Source Data 2/HA.png]

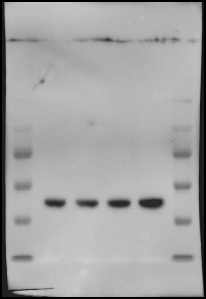

Supplement: Figure 1—figure supplement 1—source data 2. [file elife-98386-fig1-figsupp1-data2.zip › Figure 1-figure supplement 1, Source Data 2/GAPDH-C.png]

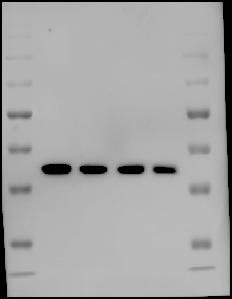

Supplement: Figure 1—figure supplement 1—source data 2. [file elife-98386-fig1-figsupp1-data2.zip › Figure 1-figure supplement 1, Source Data 2/GAPDH-E.png]

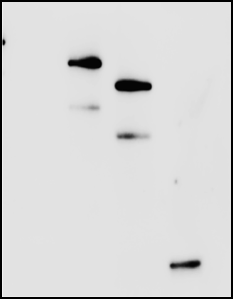

Supplement: Figure 1—figure supplement 1—source data 2. [file elife-98386-fig1-figsupp1-data2.zip › Figure 1-figure supplement 1, Source Data 2/V5-C.png]

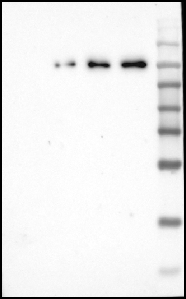

Supplement: Figure 1—figure supplement 1—source data 2. [file elife-98386-fig1-figsupp1-data2.zip › Figure 1-figure supplement 1, Source Data 2/V5-E.png]

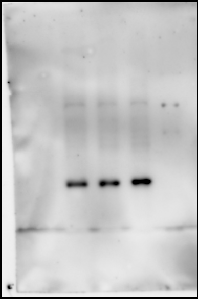

Supplement: Figure 2—source data 2. [file elife-98386-fig2-data2.zip › Figure 2, Source Data 2/FIG2A GAPDH.png]

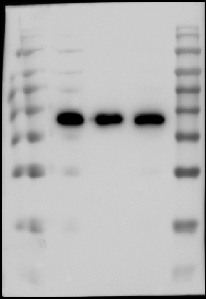

Supplement: Figure 2—source data 2. [file elife-98386-fig2-data2.zip › Figure 2, Source Data 2/FIG2A Input VGLL2-NCOA2.png]

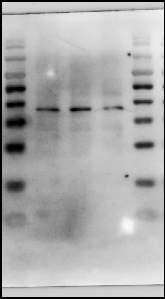

Supplement: Figure 2—source data 2. [file elife-98386-fig2-data2.zip › Figure 2, Source Data 2/FIG2A IP VGLL2-NCOA2.png]

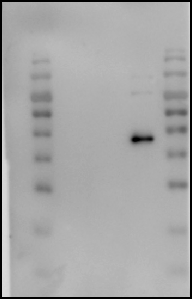

Supplement: Figure 2—source data 2. [file elife-98386-fig2-data2.zip › Figure 2, Source Data 2/FIG2A TEAD1.png]

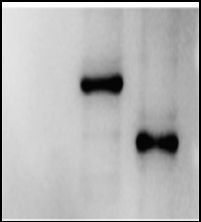

Supplement: Figure 2—source data 2. [file elife-98386-fig2-data2.zip › Figure 2, Source Data 2/FIG2A YAP5SA TEAD.tif]

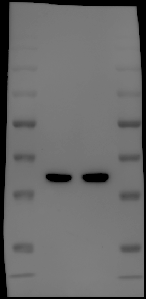

Supplement: Figure 2—source data 2. [file elife-98386-fig2-data2.zip › Figure 2, Source Data 2/FIG2B GAPDH.png]

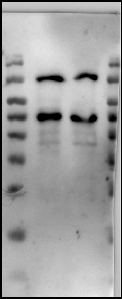

Supplement: Figure 2—source data 2. [file elife-98386-fig2-data2.zip › Figure 2, Source Data 2/FIG2B Input panTEAD.png]

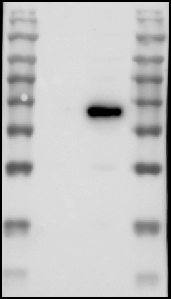

Supplement: Figure 2—source data 2. [file elife-98386-fig2-data2.zip › Figure 2, Source Data 2/FIG2B Input VGLL2-NCOA2.png]

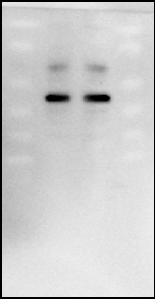

Supplement: Figure 2—source data 2. [file elife-98386-fig2-data2.zip › Figure 2, Source Data 2/FIG2B Input YAPTAZ.png]

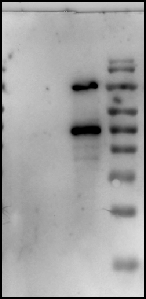

Supplement: Figure 2—source data 2. [file elife-98386-fig2-data2.zip › Figure 2, Source Data 2/FIG2B IP panTEAD.png]

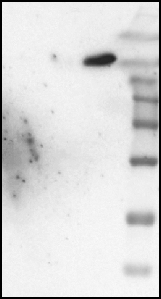

Supplement: Figure 2—source data 2. [file elife-98386-fig2-data2.zip › Figure 2, Source Data 2/FIG2B IP VGLL2-NCOA2.png]

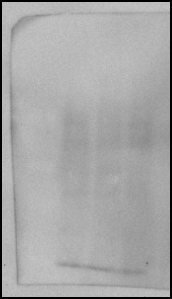

Supplement: Figure 2—source data 2. [file elife-98386-fig2-data2.zip › Figure 2, Source Data 2/FIG2B IP YAPTAZ.png]

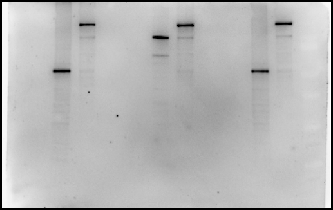

Supplement: Figure 2—source data 2. [file elife-98386-fig2-data2.zip › Figure 2, Source Data 2/FIG2C FLAG.png]

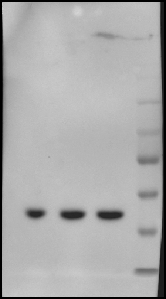

Supplement: Figure 2—source data 2. [file elife-98386-fig2-data2.zip › Figure 2, Source Data 2/FIG2C GAPDH.png]

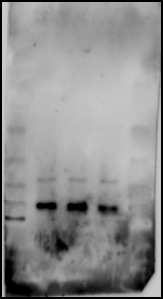

Supplement: Figure 2—source data 2. [file elife-98386-fig2-data2.zip › Figure 2, Source Data 2/FIG2C Input YAPTAZ.png]

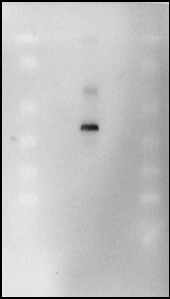

Supplement: Figure 2—source data 2. [file elife-98386-fig2-data2.zip › Figure 2, Source Data 2/FIG2C IP YAPTAZ.png]

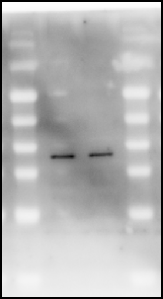

Supplement: Figure 2—source data 2. [file elife-98386-fig2-data2.zip › Figure 2, Source Data 2/FIG2F GAPDH.png]

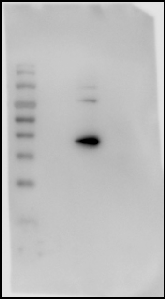

Supplement: Figure 2—source data 2. [file elife-98386-fig2-data2.zip › Figure 2, Source Data 2/FIG2F TEAD-ENR.png]

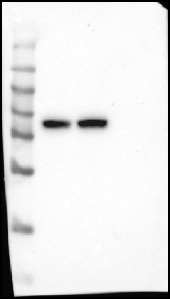

Supplement: Figure 2—source data 2. [file elife-98386-fig2-data2.zip › Figure 2, Source Data 2/FIG2G GAPDH.png]

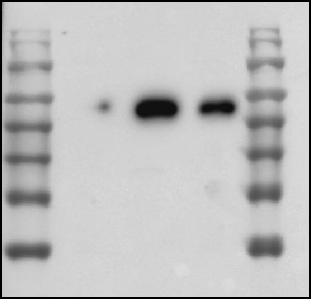

Supplement: Figure 2—source data 2. [file elife-98386-fig2-data2.zip › Figure 2, Source Data 2/FIG2G Input VGLL2-NCOA2.png]

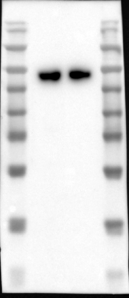

Supplement: Figure 2—source data 2. [file elife-98386-fig2-data2.zip › Figure 2, Source Data 2/FIG2G IP VGLL2-NCOA2.png]

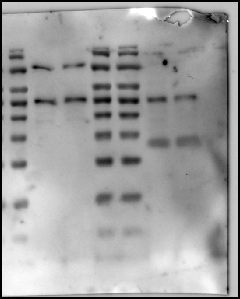

Supplement: Figure 2—source data 2. [file elife-98386-fig2-data2.zip › Figure 2, Source Data 2/FIG2G panTEAD.png]

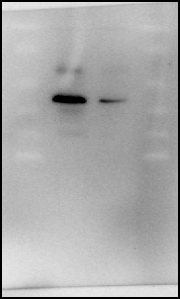

Supplement: Figure 2—source data 2. [file elife-98386-fig2-data2.zip › Figure 2, Source Data 2/FIG2G YAPTAZ.png]

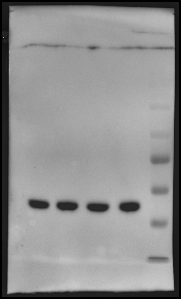

Supplement: Figure 4—source data 2. — The molecular weight markers are indicated. [file elife-98386-fig4-data2.zip › Figure 4, Source Data 2/GAPDH.png]

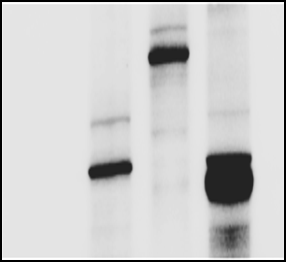

Supplement: Figure 4—source data 2. — The molecular weight markers are indicated. [file elife-98386-fig4-data2.zip › Figure 4, Source Data 2/Input FLAG.png]

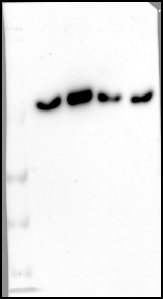

Supplement: Figure 4—source data 2. — The molecular weight markers are indicated. [file elife-98386-fig4-data2.zip › Figure 4, Source Data 2/input p300.png]

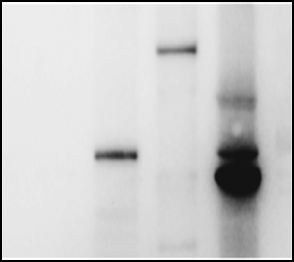

Supplement: Figure 4—source data 2. — The molecular weight markers are indicated. [file elife-98386-fig4-data2.zip › Figure 4, Source Data 2/IP FLAG.png]

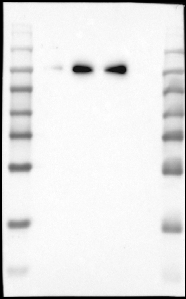

Supplement: Figure 4—source data 2. — The molecular weight markers are indicated. [file elife-98386-fig4-data2.zip › Figure 4, Source Data 2/IP p300.png]

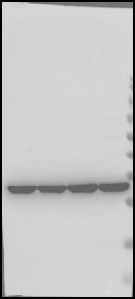

Supplement: Figure 5—source data 2. — The molecular weight markers are indicated. [file elife-98386-fig5-data2.zip › Figure 5, Source Data 2/GAPDH.png]

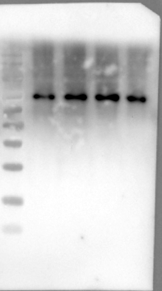

Supplement: Figure 5—source data 2. — The molecular weight markers are indicated. [file elife-98386-fig5-data2.zip › Figure 5, Source Data 2/Input p300.png]

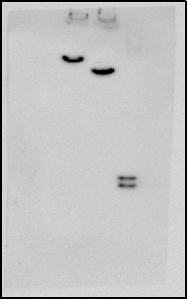

Supplement: Figure 5—source data 2. — The molecular weight markers are indicated. [file elife-98386-fig5-data2.zip › Figure 5, Source Data 2/Input V5.png]

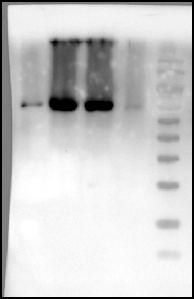

Supplement: Figure 5—source data 2. — The molecular weight markers are indicated. [file elife-98386-fig5-data2.zip › Figure 5, Source Data 2/IP p300.png]

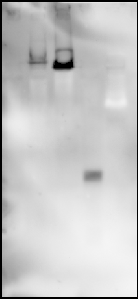

Supplement: Figure 5—source data 2. — The molecular weight markers are indicated. [file elife-98386-fig5-data2.zip › Figure 5, Source Data 2/IP V5.png]

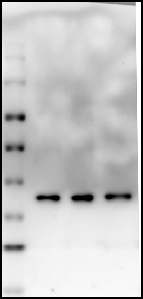

Supplement: Figure 6—source data 2. — The molecular weight markers are indicated. [file elife-98386-fig6-data2.zip › Figure 6, Source Data 2/GAPDH.png]
