# Supplementary material for: VGLL2 and TEAD1 fusion proteins identified in human sarcoma drive YAP/TAZ-independent tumorigenesis by engaging EP300
Source: eLife. 2025 May 8;13:RP98386. doi: 10.7554/eLife.98386 (PMC12061476; doi:10.7554/eLife.98386)
Supplement: Figure 2—source data 1. — The molecular weight markers are indicated. [file elife-98386-fig2-data1.zip › Figure 2, Source Data 1.pdf]

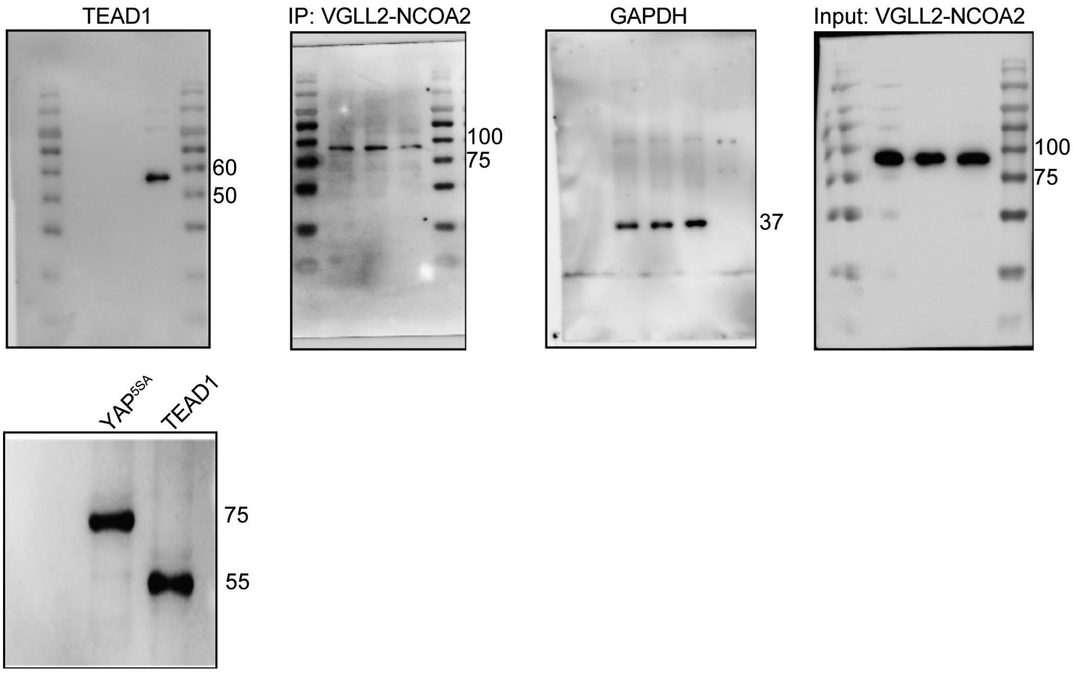

TEAD1
60
50
IP: VGLL2-NCOA2
100
75
GAPDH
37
Input: VGLL2-NCOA2
100
75
YAP5SA
TEAD1
75
55

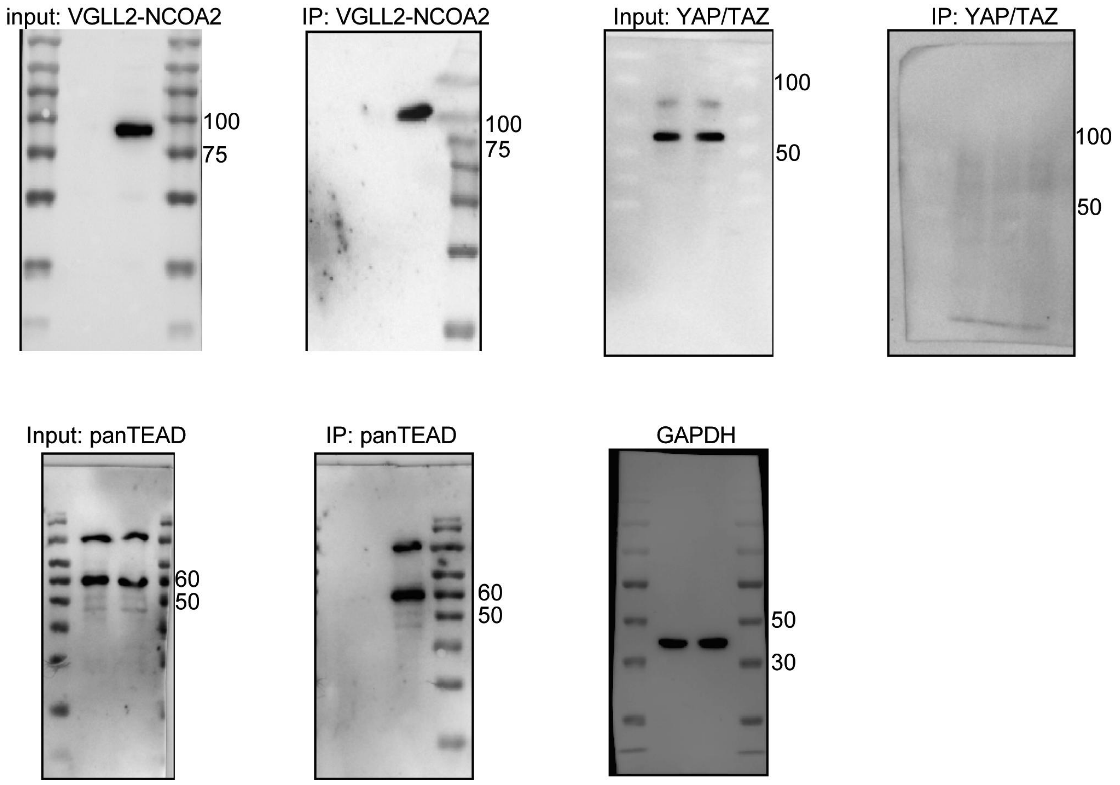
input: VGLL2-NCOA2
100
75
IP: VGLL2-NCOA2
100
75
Input: YAP/TAZ
100
50
IP: YAP/TAZ
100
50
Input: panTEAD
60
50
IP: panTEAD
60
50
GAPDH
50
30

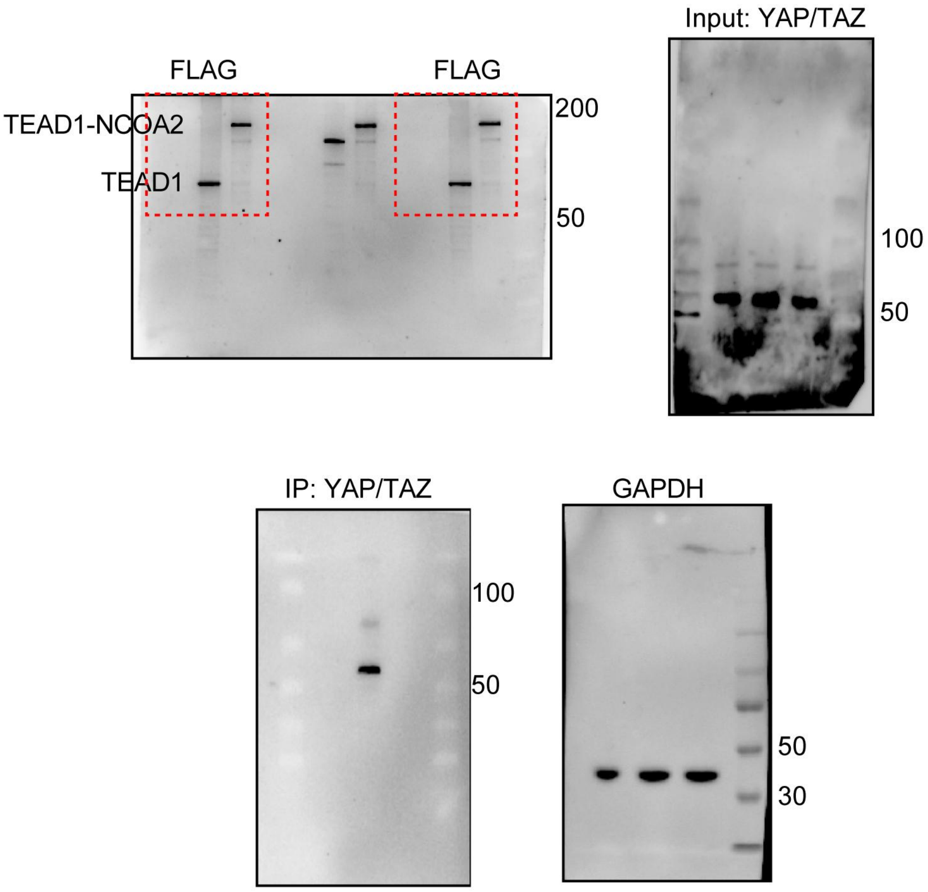
FLAG
FLAG
TEAD1-NCOA2
TEAD1
200
50
Input: YAP/TAZ
100
50
IP: YAP/TAZ
100
50
GAPDH
50
30

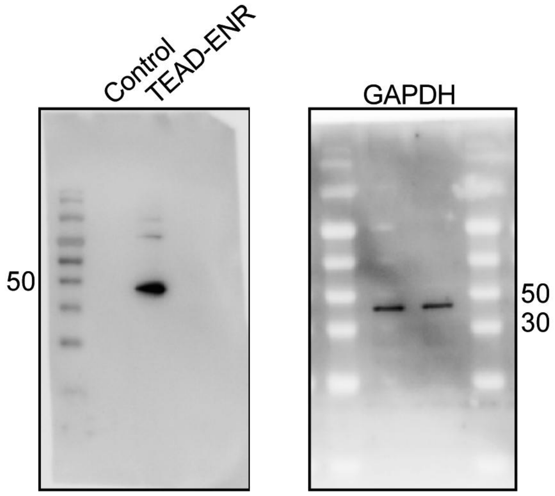
Control
TEAD-ENR
50
GAPDH
50
30

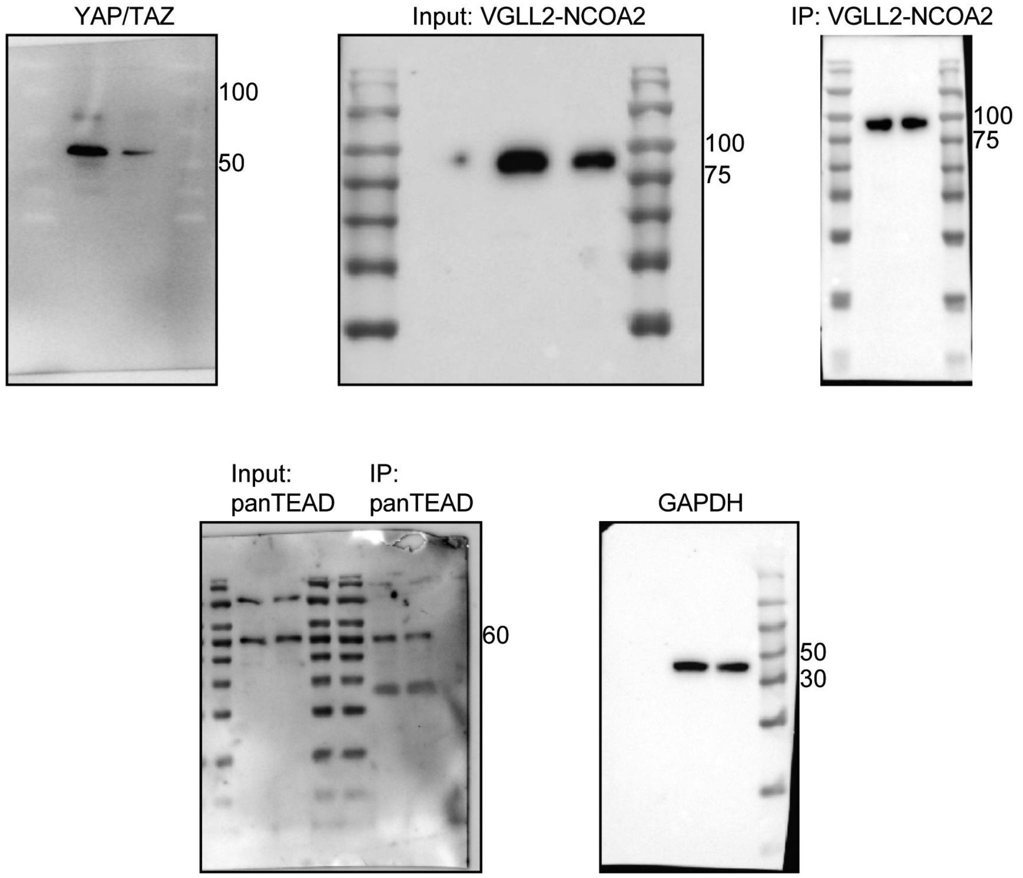

Figure 2, Source Data 1. Original western blot membranes corresponding to Figure 2A, 2B, 2C, 2F and 2G indicating the relevant bands. The molecular weight markers are indicated.
