# Supplementary material for: VGLL2 and TEAD1 fusion proteins identified in human sarcoma drive YAP/TAZ-independent tumorigenesis by engaging EP300
Source: eLife. 2025 May 8;13:RP98386. doi: 10.7554/eLife.98386 (PMC12061476; doi:10.7554/eLife.98386)
Supplement: Figure 5—source data 1. — The molecular weight markers are indicated. [file elife-98386-fig5-data1.zip › Figure 5, Source Data 1.pdf]

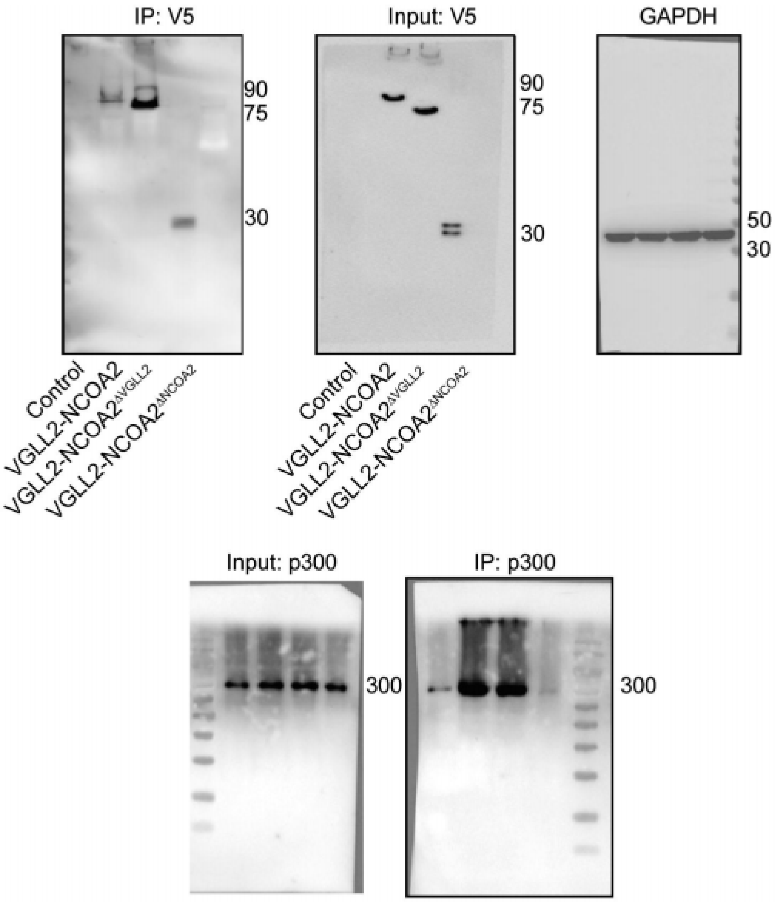

Figure 5, Source Data 1. Original western blot membranes corresponding to Figure 5A indicating the relevant bands. The molecular weight markers are indicated.
